# Standardizing computed tomographic assessment of the mesopancreas in pancreatic cancer patients

**DOI:** 10.1007/s00261-026-05486-1

**Published:** 2026-04-09

**Authors:** Stephan David, Sami Safi, Bijan Fink, Iskender Pustu, Sascha Vaghiri, Ahmad Sultani, Andrea Alexander, Michael Wolf-Vollenbroeker, Lena Haeberle-Graser, Christoph Roderburg, Irene Esposito, Wolfram Knoefel, Farid Ziayee

**Affiliations:** Heinrich Heine University Düsseldorf, Düsseldorf, Germany

**Keywords:** HPDAC, Mesopancreas, Fat stranding, Radiographic imaging, MDCT

## Abstract

**Purpose:**

The mesopancreas (MP), the dorsal area of the pancreas, is infiltrated in the majority of patients with pancreatic ductal adenocarcinoma of the pancreatic head (hPDAC) and is associated with an increased risk of incomplete resection. Current radiographic resectability criteria primarily focus on tumor involvement of major peripancreatic vessels. However, the dorsal resection margin, represented by the mesopancreas, is not explicitly considered in current staging concepts. The aim of this study was therefore to standardize a quantitative radiographic assessment of the mesopancreas and to correlate these findings with histopathology.

**Methods:**

Radiographic parameters of the primary tumor and the mesopancreas were evaluated in 173 patients with hPDAC who underwent upfront surgery. Patients were stratified according to NCCN anatomical resectability criteria. Mesopancreatic dimensions (mm) and density (HU) were analyzed on preoperative multidetector computed tomography (MDCT). Nineteen patients served as a reference group. Histopathological evaluation followed the Leeds Pathology Protocol (LEEPP).

**Results:**

124 patients (71.7%) were classified as primary resectable and 49 (28.3%) as borderline resectable. Mesopancreatic infiltration was present in 125 patients (72.3%). Resectability classification was not associated with mesopancreatic infiltration (*p* = 0.344; OR 1.5). The median density of the mesopancreas in hPDAC patients was 3 HU. Increased mesopancreatic density correlated with histopathologically confirmed mesopancreatic infiltration (*p* = 0.005), with an optimal threshold of −9 HU. In subgroup analysis, this association remained significant in primary resectable patients (*p* = 0.021) but not in borderline resectable tumors (*p* = 0.780). Additionally, higher mesopancreatic density was associated with poorer disease-free survival in primary resectable patients (*p* = 0.040).

**Conclusion:**

Increased mesopancreatic density on preoperative MDCT correlated with histopathologically confirmed mesopancreatic tumor infiltration. Radiographic assessment of mesopancreatic density may therefore provide additional information for preoperative risk stratification.

**Supplementary Information:**

The online version contains supplementary material available at https://doi.org/10.1007/s00261-026-05486-1.

## Introduction

Multi-detector computed tomography (MDCT) of the abdomen remains the gold standard in preoperative diagnostics and staging for pancreatic ductal adenocarcinoma of the pancreatic head (hPDAC). Despite improvements in margin-negative (R0) resection rates in patients with borderline resectable PDAC following the introduction of neoadjuvant treatment strategies, reported R0 rates for the overall PDAC population remain relatively low [1, 2]. Population-based analyses have even reported decreasing R0 rates over time, likely due to increased pathological awareness and stricter assessment of resection margins [3]. This highlights the importance of achieving circumferential margin-negative resection (R0CRM−), which is strongly associated with improved survival [4–6], and underscores the need for improved preoperative risk stratification to identify patients most likely to achieve complete circumferential tumor clearance.

Current radiographic resectability criteria primarily focus on tumor involvement of major peripancreatic vessels, particularly within the medial vascular groove [7]. However, the dorsal resection margin remains at comparable risk for a R1 resection [8] but is not considered during preoperative staging [9]. We recently highlighted the benefit of mesopancreatic excision (MPE) during structured pancreatoduodenectomy for hPDAC patients to secure the dorsal resection margin and its fascial integrity as well as the cranio- and caudomedial aspects [10, 11].

The mesopancreatic area remains a matter of ongoing debate and comprises the dorsolateral and -medial boundaries of the duodenum and pancreas, extending to the medial anchor point around the superior mesenteric artery (SMA) and coeliac trunk (TC); an area that is currently only considered during staging in borderline- or non-resectable patients if a direct tumor contact with the SMA/TC is present [7]. Contemporary anatomical text books from the end and beginning of the 19th and 20th century respectively have already mentioned these principles [12–14]. Over the last century however the main surgical focus was to standardize pancreatic surgery instead of targeting its radicality. Only a few surgeons have propagated a radical approach without demonstrating a significant survival benefit [15]. Previous overestimated rates of R0 resections further contributed to the low motivation to improve local tumor control [16].

The incorporation of anatomical landmarks into surgical resection strategies is not a novel concept and has already been standardized by Heald and others [17–19].

We previously demonstrated that in over 78% of the hPDAC patients, the mesopancreas was infiltrated [10]. We confirmed these findings in a consecutive cohort of patients treated with upfront surgery [10] and verified this on a neoadjuvant treated subgroup [20]. Upfront resected hPDAC patients with a localized disease, in whom an invasion to the portomesenteric system and arterial vasculature was histopathologically ruled out, remained at risk for mesopancreatic infiltration [11]. Taken together, the available evidence suggests that the infiltration status of mesopancreas may considered additionally during preoperative resectability assessment [9].

We and others previously were able to demonstrate the feasibility of computed tomographic fat stranding in the mesopancreas, to identify those hPDAC patients with mesopancreatic infiltration [20–23]. However, such signs are observer dependent and based upon a subjective scoring without an established reference standard. The aim of this study is to provide a quantitative and therefore objective analysis of the mesopancreas and implement radiomorphologic parameters for a standardized description of the mesopancreas in hPDAC patients.

## Materials and methods

### Demographic data

A total of 173 consecutively treated hPDAC patients were included, who underwent upfront surgery for primary or borderline resectable hPDAC at the University Hospital of Duesseldorf between September 2015 and January 2024. All non-metastasized patients who received upfront surgery were included. Exclusion criteria were: CT imaging outside our institution, CT images with insufficient quality (slice thickness > 2 mm). All preoperative CT scans were performed prior to surgery. To avoid bias from neoadjuvant treatment-related tissue changes such as fibrosis or scarring, patients who had received neoadjuvant therapy were excluded. Although some patients fulfilled criteria for borderline resectability according to our current reassessment, neoadjuvant therapy was not administered due to pronounced obstructive jaundice, persistent cholangitis, or patient preference. A preoperative interdisciplinary tumor board decision was made for all patients. Furthermore, we established a control group of 19 patients with hepatocellular carcinoma (HCC), who received a MDCT of the abdomen for follow-up purposes and had no proven pancreatic pathology.

### Radiographic imaging

All examinations were performed on three multi-detector CT systems at our hospital- SOMATOM Edge (128 × 0.6 mm), SOMATOM Definition Flash (128 × 0.6 mm), and SOMATOM Definition AS (64 × 0.6 mm) (all Siemens Healthineers) – following our two-phase standard operating procedure (SOP). Collimation and primary reconstructions were harmonized across scanners (axial 2 mm soft-tissue, axial 1 mm lung for thorax, axial 2 mm bone; with 3 mm coronal/sagittal MPRs). Dose modulation (CARE Dose4D) was enabled on all scanners. Tube voltage for contrast phases was fixed at 100 kV on all systems; topogram and monitoring runs used 120 kV. Rotation time was 0.28–0.50 s depending on device. Reconstruction used vendor iterative reconstruction (SAFIRE/ADMIRE, strength 3) with medium-smooth kernels for soft tissue and standard lung kernel for 1 mm lung images. Contrast agent and injection. Iohexol 300 mg I/mL (Accupaque 300), 80 mL (≥ 95 kg: 90 mL), followed by 30 mL saline chaser; injection rate 3 mL/s via 18–20G antecubital line. Primary reconstructions and window/level settings were standardized as in the SOP (soft tissue 2 mm W/L 350/50 HU; lung 1 mm W/L 1500/−500 HU; bone 2 mm W/L 2000/500 HU; coronal and sagittal MPRs at 3 mm). CT-acquisition parameters were harmonized across all scanners. Contrast-enhanced phases were acquired at 100 kV, whereas the topogram and monitoring scans were performed at 120 kV. Tube current modulation (CARE Dose4D) was applied using scanner-specific quality reference mAs values. For the upper-abdominal arterial spiral, reference settings typically ranged between 95 and 125 mAs, while thoracoabdominal portal-venous acquisitions consistently used approximately 160–170 mAs across all devices. Rotation times varied between 0.28 and 0.5 s with a pitch of 0.8–1.2, according to device-specific defaults, which were maintained to ensure comparable image noise and radiation dose. Collimation was 128 × 0.6 mm on the Flash and Edge systems and 64 × 0.6 mm on the Definition AS, with primary reconstructions at 2 mm for soft tissue and 1 mm for lung. All reconstructions used vendor-specific iterative algorithms (ADMIRE/SAFIRE, strength levels 3–4), applying harmonized kernel families for soft tissue (I30f/Br36f equivalents), lung (Br64f), and bone (B70f/B75f). All helical scans were performed during an end-inspiratory breath-hold.

These settings were held constant across scanners to ensure quantitative comparability of Hounsfield-unit (HU) measurements in the mesopancreas; only rotation time and pitch varied within the above limits to accommodate scanner geometry. The SOP coverage, phase timing, and reconstruction planes are identical across devices. All measurements were performed on Sectra IDS7 PACS using the standard polygon ROI tool and caliper tools.

**Fig. 1:**
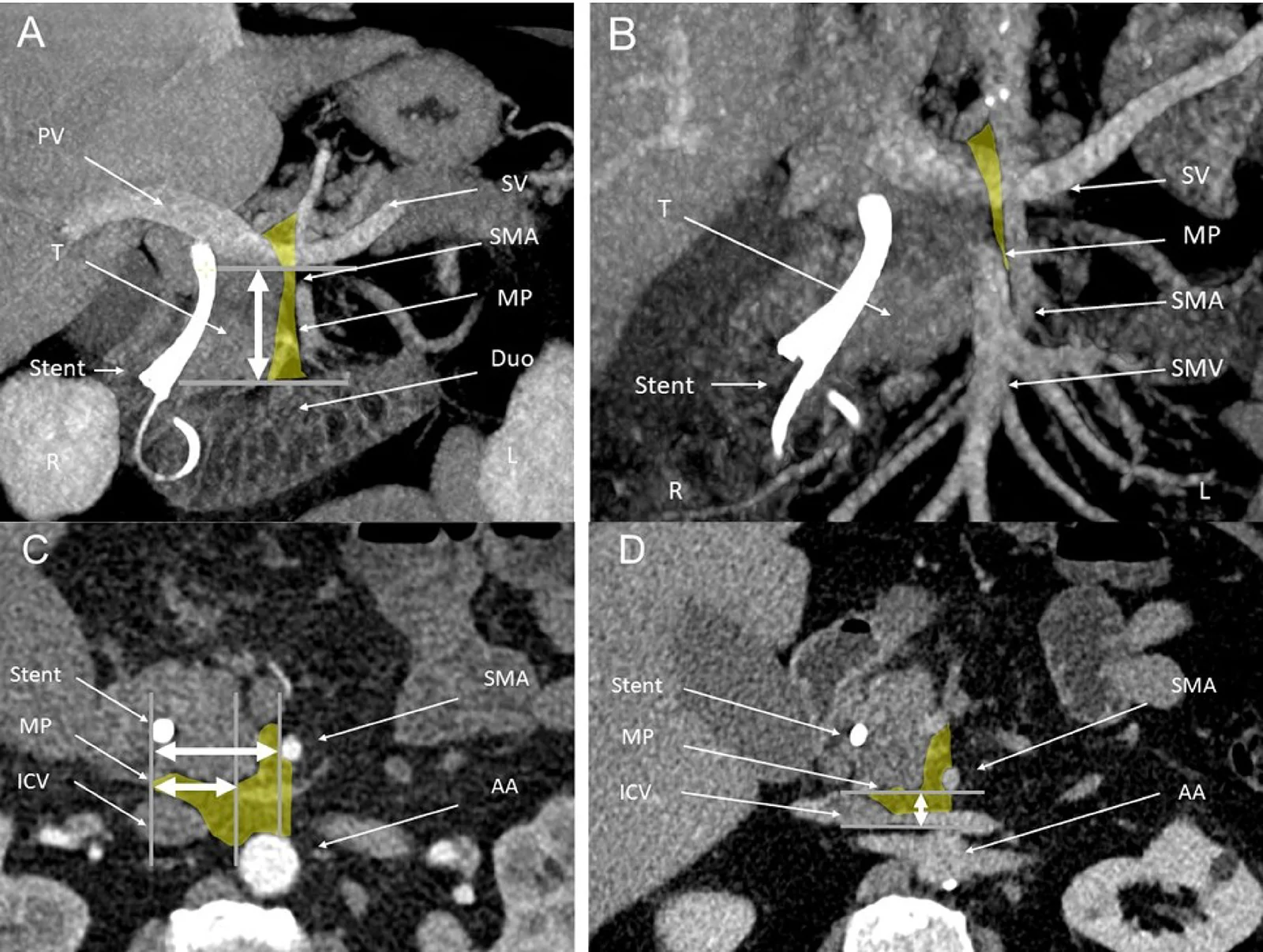
CT-scan measurements provide a standardized approach for quantitatively evaluating mesopancreatic dimensions and density using preoperative imaging: **A** Cranio-caudal extension in coronary layer: Measured from the caudal border of the portal vein to the cranial border of the pars horizontalis duodeni, tangential to the medial border of the pars descendens duodeni (double arrowed line). **B** Coronary visualization of the mesopancreas in reference to the major peripancreatic vessels. Note the anatomical space between the pancreatic tissue and the SMA (yellow hatched area) **C** Horizontal-SMA-extension in transversal CT layer. (1) Horizontal-SMA extension: Measured from the medial border of the pars descendens duodeni to the right border of the superior mesenteric artery, in the last axial layer of the pars descendens duodeni (double arrowed line). Horizontal-AA extension: From the medial border of the pars descendens duodeni to the right border of the abdominal aorta (double arrowed line). **D** Dorso-ventral-tumor extension and Hounsfield measurement of the pancreas in transversal CT layer: Between the dorsal edge of the pancreatic head and the abdominal aorta/inferior caval vein with an auxiliary line, in the layer with the greatest tumor extension (double arrowed line). Furthermore measurements of the Hounsfield unit density in the mesopancreas (yellow hatched area): The average density of the mesopancreas, measured in Hounsfield units, was assessed across the entire region at the greatest pancreatic head extension in the control group or at the level of maximum tumor extension in the hPDAC group. (*Duo* duodenum, *MP* mesopancreas (yellow hatched area), *PV* portal vein, *SMA* superior mesenteric artery, *SMV* superior mesenteric vein, *SV* splenic vein, *T* tumor; grey lines = auxiliary lines; double arrowed line = measured extension)

**Fig. 2:**
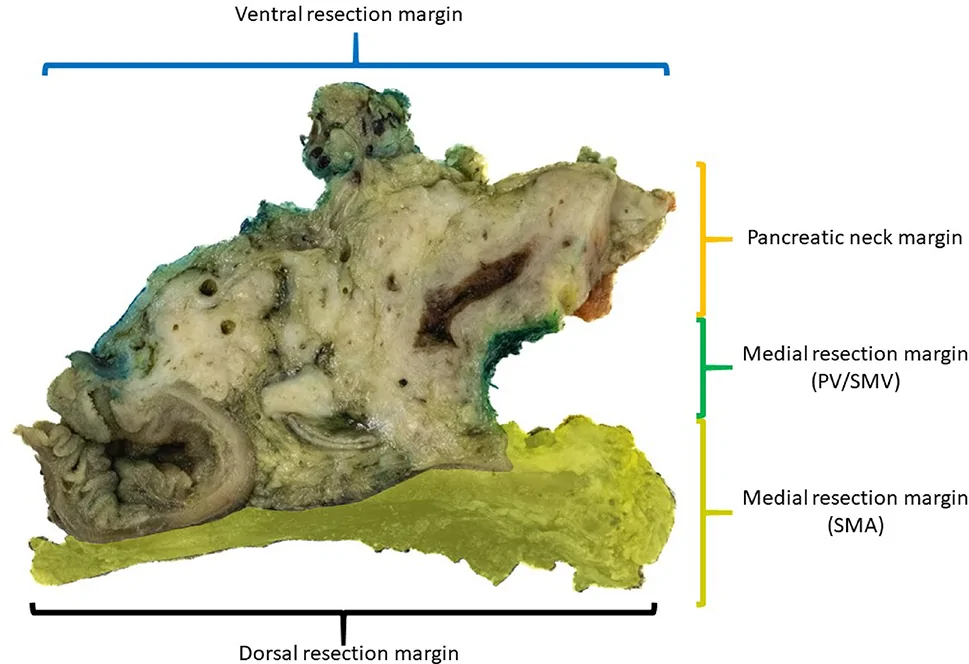
The pathological specimen after pancreatoduodenectomy exhibits invasion into the adjacent peripancreatic adipose tissue (pT2N1L1V1Pn1R0CRM−). To aid in accurate anatomical delineation, the specimen was meticulously marked using a standardized color-coding protocol (dorsal margin = black; ventral margin = blue; medial PMV/SMV margin = green; medial SMA margin = yellow; mesopancreas = yellow hatched area). Macroscopic examination was performed following the axial slicing method ensuring a thorough and methodical assessment of the tissue structure

The area of the mesopancreas was determined in interdisciplinary consensus with colleagues of the Department of Anatomy of the Heinrich Heine University Duesseldorf. Historically it comprises the area between the pancreatic tissue and the Treitz fascia and extends towards the superior mesenteric artery [12, 14, 24]. The ventral margin of the mesopancreas corresponds to the pancreatic tissue. The dorsal margin is delineated by the inferior vena cava and the abdominal aorta, and the medial margin by the superior mesenteric artery (Figs. 1 and 2). The portomesenteric system has a different embryologic origin than the pancreas and is located ventromedial to the mesopancreas. The ventral fascial envelope does not cover the portomesenteric system and therefore the mesopancreas is located underneath the portal veins until it reaches the superior mesenteric artery ventrally. Figures 1A and B highlight the mesopancreas from a coronary view (yellow hatched area). Figures 1C and D highlight the mesopancreas from an axial view (yellow hatched area).

Multidetector computed tomography scans of the patients were evaluated (Supplemental Table 1) by two experienced hepatopancreatobiliary radiologists (FZ and BF) following a predefined assessment protocol. The anatomical delineation of the mesopancreas was established in interdisciplinary consensus with specialists from surgery (SAS and SD) and anatomy (M.W-V) prior to quantitative analysis, ensuring anatomically accurate and reproducible definition of the region of interest.

Based on this consensus-defined anatomical framework, mesopancreatic density (expressed in Hounsfield units) and geometric dimensions were measured using standardised, PACS-based measurement tools. In each MDCT scan of the study group and control group, the mesopancreas was analyzed and stratified according to the following geometric data (Figs. 1A-D): (1) Cranio-caudal extension of the mesopancreas measured from the caudal border of the portal vein to the cranial border of the pars horizontalis duodeni tangential to the medial border of the pars descendens duodeni (Cranio-caudal extent) (Fig. 1A). (2A) Horizontal extension measured from the medial border of the pars descendens duodeni to the right border of the superior mesenteric artery in the last layer of the pars descendens duodeni (horizontal-SMA-extent) (Fig. 1C). (2B) Horizontal extension was from the medial border of the pars descendens duodeni to the right border of the abdominal artery (horizontal-AA-extent) (Fig. 1C). (3) Dorso-ventral extension measured from the dorsal edge of the pancreatic head and an auxiliary line between the abdominal aorta and the inferior vena cava in the first layer caudal to the portal vein (dorso-ventral PV-extent). (4) Dorso-ventral extent measured from the dorsal edge of the pancreatic head and an auxiliary line between the abdominal aorta and the inferior vena cava in the layer with the greatest axial tumor extent (dorso-ventral Tumor extent) (Fig. 1D).

In addition, we performed density measurements by determining the average density in Hounsfield units inside a polygonal ROI including the entire mesopancreas (Yellow hatched area in Fig. 1C and D).

### Histopathological analysis

The histopathological prepared samples of the hPDAC patients were taken from the pathological reports. In particular, carcinomatous mesopancreatic fat infiltration was assessed as a binary variable (yes/no) during histopathological staging. All hPDAC patients included in this study were evaluated according to the concept of circumferential resection margins (CRM) and to the recommendations of the *Royal College of Pathologists* [8, 25–28] (Fig. 2).

### Surgical therapy

All resections were performed by surgeons experienced in hepato-pancreato-biliary surgery. During mesopancreatic excision, mobilisation of the pancreatic head was performed along the Treitz and Fredet fascia [10, 11]. The purpose is to entirely excise the lymphatic and nerve-associated tissues adjacent to the pancreatic head and the uncinate process to secure local tumor control.

### Statistical analysis

Numerical data were analyzed using the Mann–Whitney U test and Pearson correlation analysis. Categorical variables were assessed using the chi-squared test. Binary logistic regression analysis was applied to identify predictive factors, with significant findings reported using odds ratios and corresponding confidence intervals. All analyses were conducted using SPSS^®^ Statistics for Windows (version 26.0; SPSS Inc., Chicago, IL, USA). A p-value < 0.05 was considered statistically significant.

The study was conducted in accordance with Good Clinical Practice guidelines and the Declaration of Helsinki and received approval from the Institutional Review Board of the Medical Faculty at Heinrich Heine University Duesseldorf (IRB No. 2019- 473_1).

## Results

### Demographic data

The demographic characteristics of the study cohort are summarised in Table 1. In all 173 hPDAC patients, the preoperative resectability status, the CRM status and the infiltration status of the mesopancreas were available for analysis. The median age at the time of treatment was 69 years (range 34–90 years); 101 patients were male (58.4%) and 72 female (41.6%). In 48 (27.7%) patients the mesopancreas (MP) was free of cancerous invasion, whereas in 125 (72.3%) patients the MP was infiltrated. According to the current resectability criteria, 124 patients (71.7%) were classified as primary resectable and 49 (28.3%) as borderline resectable. Since the final resection margin status depends on the status of each resection margin, we computed the dorsal as well as the medial resection margins separately (Table 1). The median age of the 19 control patients was 67 years (range 45–78 years), 7 of them were female (36.8%).

**Table 1:** Demographic data of all 173 patients

Age in years		
Median (range)	69 (34–90)	
Gender	n	%
Male	101	58.4
Female	72	41.6
T-stage		
T1	12	6.9
T2	89	51.4
T3	69	39.9
T4	3	1.7
N-stage		
N0	37	21.4
N1	89	51.4
N2	47	27.2
Grading		
G1	7	4.1
G2	85	49.1
G3	81	46.8
Pn		
Pn0	24	13.9
Pn1	149	86.1
L		
L0	84	48.6
L1	89	51.4
V		
V0	115	66.5
V1	58	33.5
R-status dorsal		
R1/R0CRM+	37	21.4
R0CRM-	136	78.6
R-status vascular groove		
R1/R0CRM+	45	26.1
R0CRM-	128	73.9
MP-status		
MP negativ	48	27.7
MP positiv	125	72.3
Resectability status		
Primary resectable	124	71.7
Borderline resectable	49	28.3

Staging is revised to the 8th edition of the UICC TNM classification of malignant Tumors. Preoperative staging classification was adopted from the NCCN

*CRM *circumferential resection margin, *MP-status* mesopancreatic infiltration status, *Pn* perineural invasion, *V* venous invasion

## hPDAC vs. reference group

**Fig. 3:**
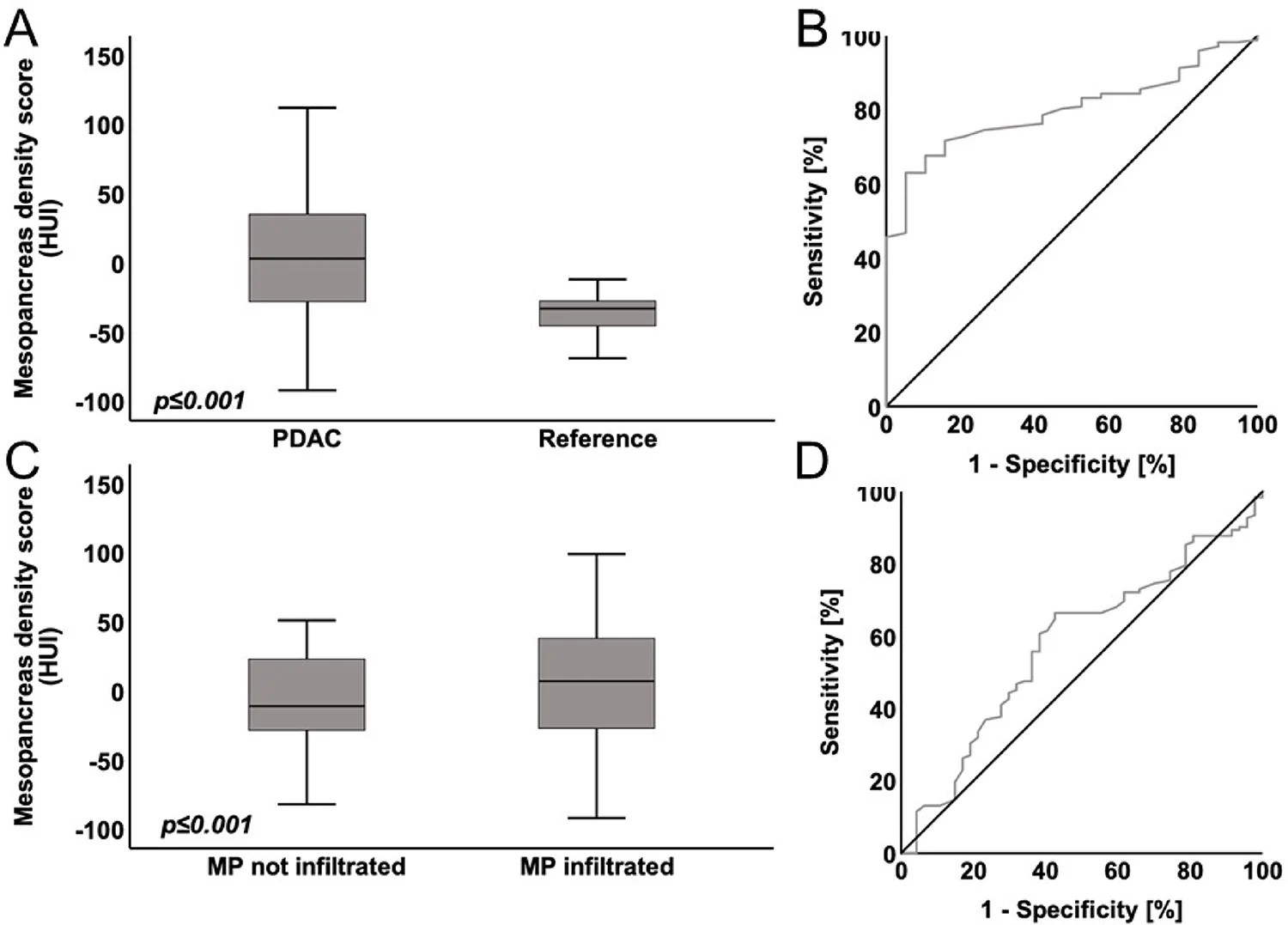
**A** Box plot analysis of radiographically assessed mesopancreatic density in hPDAC patients vs. reference group. Mann-Whitney U test was used to test for statistical difference between these groups. **B** ROC analysis for mesopancreatic density, hPDAC patients vs. reference group. **C** Box plot analysis of radiographically assumed mesopancreatic density in relation to the true mesopancreatic infiltration status. Mann-Whitney U test was used to test for statistical significance. **D** ROC analysis for mesopancreatic density vs. true mesopancreatic infiltration status in hPDAC patients

The radiological data for the hPDAC cohort and the reference group are shown in the supplemental Table 2. All geometric variables were homogeneously distributed between the two groups (Supplemental Table 2), indicating comparable anatomical conditions. We concluded a selection in both study cohorts. The median density of the entire mesopancreas at the area of the greatest tumor extension in the hPDAC cohort was 3.0 HU (−92.0–112.0.0.0 HU) and significantly higher when compared to the reference group (median HU score = − 33.0 HU (−82 − 7.0 HU) (p < 0.001) (Supplemental Tables 2 and Fig. 3A). When hPDAC patients were stratified according to current resectability criteria and primary and borderline resectable patients were analyzed separately compared to the reference group, no additional significant differences were observed.

Through the ROC analysis we identified an optimal cut-off of −11.5HU to differentiate between hPDAC patients and patients without a pancreatic pathology (Supplemental Tables 2 and Fig. 3B). According to this value we sub-grouped the patients with a density at the MP < − 11.5HU and ≥ − 11.5HU and computed them against the state of disease (hPDAC vs. reference) (*p < 0.001*) (Supplemental Table 3). Logistic regression analysis demonstrated a 30.7-fold increased likelihood of hPDAC when mesopancreatic density exceeded −11.5 HU (Supplemental Table 3).

### Resectability status according to NCCN vs. dimensions and density score in the hPDAC group

In order to evaluate if the current resectability criteria predict the mesopancreatic infiltration status, we sub-grouped all 173 hPDAC patients according to the current radiographic standards (Table 1 and Supplemental Table 4). All radiographically assessed geometric dimensions as well as mesopancreatic density were homogeneously distributed between primary and borderline resectable patients.

Neither ROC analysis nor predictive modelling yielded statistically significant results, indicating that current resectability status failed to predict mesopancreatic infiltration (Supplemental Table 5).

### MP status vs. dimensions and density in the hPDAC group

The average density of the entire mesopancreas at the level of the greatest tumor extension was analyzed for every patient in the hPDAC group. hPDAC patients with an infiltration of the mesopancreas showed a significantly increased density score when compared to MP- hPDAC patients (*p* < 0.001) (Table 2; Fig. 3C). All dimensions were homogeneously distributed between the MP + and MP− hPDAC patients (Table 2). ROC analysis identified an optimal cut-off value of −9.0 HU to discriminate between MP + and MP− patients (Table 3; Fig. 3D). Stratification using this cut-off (< − 9 HU vs. ≥ −9 HU) demonstrated a significant association with histopathologically confirmed mesopancreatic infiltration (*p* = 0.005) (Table 3). Logistic regression analysis revealed a 2.7-fold increased risk of mesopancreatic infiltration in patients with mesopancreatic density ≥ − 9.0 HU (Table 3).

**Table 2:** Correlation analysis between mesopancreas infiltration status and geometric-extension data as well as MP density score within in the hPDAC group

	Mesopancreas infiltrated *n* = 125	Mesopancreas not infiltrated *n* = 48	
	Median (min.-max.)	Median (min.-max.)	*p*-value
Cranio- caudal extent (mm)	59.0 (40.0–90.0)	65.0 (34.0–95.0)	*0.113*
Horizontal-SMA-extent (mm)	29.0 (7.0–63.0)	31.0 (2.0–54.0)	*0.066*
Horizontal-AA-extent (mm)	36.0 (16.0–67.0)	37.0 (9.0–71.0)	*0.667*
Dorso-ventral-extent-PV (mm)	13.0 (2.0–28.0)	11.0 (4.0–28.0)	*0.347*
Dorsal-ventral-T-extent- (mm)	8.0 (2.0–26.0)	6.0 (2.0–230.0.)	*0.284*
Density-P (HU)	7.0 (−92.0–99.0)	−11.0 (−82.0–112.0)	***< 0.001***

Statistical significance was calculated using the Mann-Whitney U test. (*p* ≤ 0.05 is significant). P-values are presented in italics, and statistically significant values are highlighted in bold

**Table 3:** Prediction analysis of the mesopancreatic infiltration status by radiographically measured mesopancreatic density scores (HU) in hPDAC patients

Optimal cut off by ROC analysis		−9.0 HU	
	MP infiltration status		*p*-value
	MP not infiltrated	MP infiltrated	***0.005***
MP density score < 9HU	28	42	Sensitivity
			*66.39%*
			Specificity
			*57.45%*
MP density score ≥ 9HU	20	83	PPV
			*80.20%*
			NPV
			*39.71%*

	p-value	Odds ratio	95% CI
Binary logistic regression analysis	***0.005***	2.7	1.3–5.3

Optimal cut-off point for density score was computed by ROC-analysis. Ordinal scaling of density scores (< Cut-off and > Cut-off) was tested for distribution against MP infiltration status (Chi-Square test). Binary logistic regression analysis was used for prediction analysis. P-values are presented in italics, and statistically significant values are highlighted in bold

In sub-group analysis of primary resectable hPDAC patients, an increased mesopancreatic density remained to correlate significantly with true mesopancreatic tumor infiltration (*p = 0.021*) (Supplemental Table 6). In borderline resectable hPDAC patients, differences in mesopancreatic density did not correlate with histopathologically confirmed infiltration (*p = 0.780*) (Supplemental Table 7).

### R-status vs. density score and dimensions

Correlation analyses between geometric dimensions, mesopancreatic density, and resection margin status of the dorsal margin and vascular groove are presented in Tables 4 and 5.

**Table 4:** Correlation analysis between dorsal R-status and geometric-extension data as well as MP density score within in the hPDAC-group

	Dorsal R0CRM− *n* = 136	Dorsal R1/R0CRM+ *n* = 37	
	Median (min.-max.)	Median (min.-max.)	*p*-value
Cranio- caudal extent (mm)	64.0 (40.0–95.0)	58.0 (34.0–76.0)	***0.018***
Horizontal-SMA-extent (mm)	30.0 (2.0–63.0)	30.0 (10.0–56.0)	*0.854*
Horizontal-AA-extent (mm)	35.5 (9.0–71.0)	40.5 (9.0–60.0)	*0.285*
Dorso-ventral-extent-PV (mm)	11.0 (2.0–28.0)	14.5 (2.0–28.0)	***0.013***
Dorsal-ventral-T-extent- (mm)	6.0 (2.0–26.0)	9.0 (2.0–26.0)	***0.006***
Density-P (HU)	4.0 (−85.0–112.0.0.0)	−6.0 (−92.0–98.0)	*0.906*

Statistical significance calculated using the Mann-Whitney U test. (*p* ≤ 0.05 is significant). P-values are presented in italics, and statistically significant values are highlighted in bold

**Table 5:** Correlation analysis between R-status of the vascular groove (medial CRM), and geometric-extension data as well as MP density score within in the hPDAC-group

	Vascular groove R0CRM- *n* = 128	Vascular groove R1/R0CRM+ *n* = 45	
	Median (min.-max.)	Median (min.-max.)	*p*-value
Cranio- caudal extent (mm)	60.5 (34–95)	65 (45–95)	*0.599*
Horizontal-SMA-extent (mm)	32 (8–62)	26.5 (2–63)	***0.017***
Horizontal-AA-extent (mm)	37 (9–65)	38.5 (12–71)	*0.837*
Dorso-ventral-extent-PV (mm)	13 (2–28)	12 (9–71)	*0.841*
Dorsal-ventral-T-extent- (mm)	6 (2–26)	6.5 (2–26)	*0.810*
Density (HU)	−2 (−85–112)	8.0 (−92–51)	*0.727*

Statistical significance was calculated using the Mann-Whitney U test. (*p* ≤ 0.05 is significant). P-values are presented in italics, and statistically significant values are highlighted in bold

Density score and R-status: Mesopancreatic density did not correlate with an increased likelihood of incomplete resection (R1/R0CRM+) at either the dorsal or medial resection margin in the overall hPDAC cohort or in subgroup analyses of primary and borderline resectable patients (Tables 4 and 5; Supplemental Tables 8–11).

**Fig. 4:**
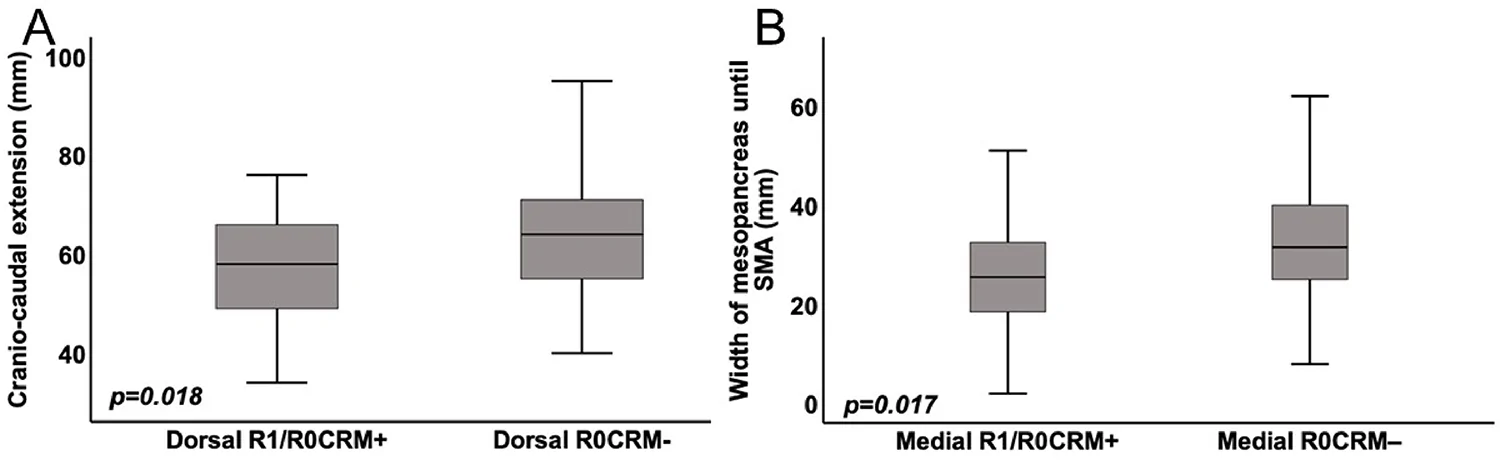
**A** Box plot analysis of radiographically assessed cranio-caudal extension of the mesopancreas vs. dorsal resection margin status. **B** Box plot analysis of radiographically assessed horizontal-SMA extension of the mesopancreas vs. medial resection margin status. Mann-Whitney U test was used to test for statistical significance

Dorsal resection margin: A reduced cranio-caudal extension (shortened length of the mesopancreas) was associated with an increased likelihood of R1/R0CRM+ status at the dorsal resection margin (*p = 0.018*) (Table 4; Fig. 4A). This association remained statistically significant in subgroup analysis with only primary resectable hPDAC patients (*p = 0.029*) (Supplemental Table 8). With regard to the dorso-ventral extension variables (depth of the mesopancreas) was counterintuitive (Table 4). A larger depth correlated with an increased likelihood of incomplete resection (R1/R0CRM+) (*p = 0.013* and *p* = 0.006). The difference was of no statistical significance in primary resectable patients (Supplemental Table 8) but remained significant in the borderline resectable hPDAC sub-group (Supplemental Table 9). All other geometric variables were homogenously distributed across the resection status of the dorsal resection margin (Table 4, Supplemental Tables 8 and 9).

Medial resection margin: A decreased horizontal extension towards the SMA (reduced mesopancreas width) correlated with an increased risk of R1/R0CRM+ status at the medial resection margin in the entire cohort (*p = 0.017*) (Fig. 4B; Table 5). All other radiographic computed variables were again homogenously distributed across the resection status of the vascular groove (Table 5). In subgroup analysis of the primary resectable hPDAC patients none of the radiographically assessed variables correlated with the medial resection margin status (Supplemental Table 10).

### Mesopancreatic density and disease-free survival

**Fig. 5:**
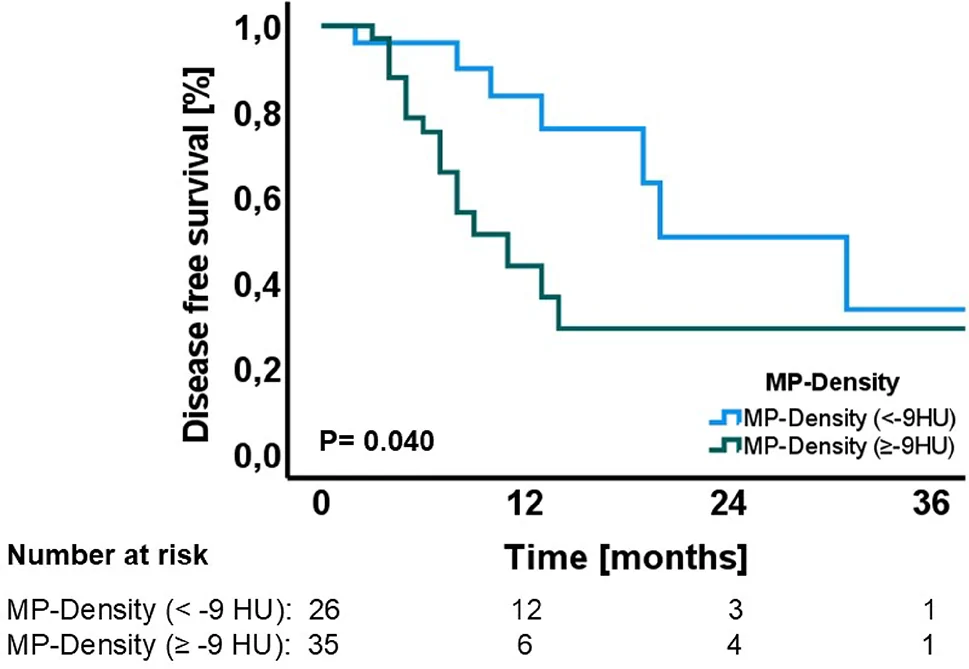
Kaplan–Meier analysis of disease-free survival (DFS) in primary resectable hPDAC patients stratified by mesopancreatic (MP) density on preoperative MDCT using the predefined cut-off of −9 HU. Patients with reduced MP density (< − 9 HU) showed significantly longer DFS compared with those with increased MP density (≥ − 9 HU) (*p = 0.040*). Numbers at risk are shown below the plot

Follow-up data for disease-free survival (DFS) were available in 61 primary resectable hPDACs. The mean follow-up duration was 22.6 months. Patients were stratified according to mesopancreatic density using the predefined cut-off value of −9 HU. Kaplan–Meier analysis demonstrated a significant difference in DFS between patients with low mesopancreatic density (< − 9 HU, median DFS = 31 months) and those with increased density (≥ − 9 HU, median DFS = 11 months). Patients in the high-density group experienced earlier disease recurrence, resulting in a significantly shorter DFS (*p = 0.040*) (Fig. 5).

A total of 23 disease recurrences occurred during follow-up. The most common site of recurrence was the liver (*n* = 12), followed by pulmonary metastases (*n* = 5). Peritoneal metastases were observed in five patients, while locoregional recurrence occurred in two cases. Recurrence events were more frequently observed among patients with increased mesopancreatic density. No statistically significant difference in DFS was observed for the entire hPDAC cohort or in the subgroup of borderline resectable patients.

## Discussion

Preoperative MDCT continues to be the gold standard for the diagnosis and resectability assessment in pancreatic cancer patients [7]. To date, the clinical focus remains on the medial resection site, particularly tumor involvement of the portomesenteric vascular system [9]. From an oncological point of view this focus is justified, since infiltration of the portomesenteric system is associated with an aggressive oncological profile and impaired systemic disease control [9]. From a surgical perspective, local tumor control is equally important [6] and all facets of tumor extensions have to be surgically and radiographically considered and assessed. We know that the dorsal resection margin, which is represented by the mesopancreas [10, 11, 29], is at a comparable risk of incomplete resection [8] when compared to the medial vascular groove. In order to focus on optimal local tumor control and the achievement of complete circumferential (R0CRM−) resection [6] equal attention must be paid to both the dorsal mesopancreatic margin and the medial vascular margin [8, 10, 11].

A similar concept exists in rectal cancer, where preoperative imaging of mesorectal infiltration and suspected mrCRM+ status guides the use of neoadjuvant treatment aiming to maximize the likelihood of complete (R0) resection and to enhance long-term survival [30–32]. By analogy, a reliable radiographic assessment of mesopancreatic infiltration could serve as an important tool for preoperative stratification in pancreatic cancer. Before such an approach can be implemented, it is first necessary to demonstrate that mesopancreatic infiltration can be reliably detected by preoperative CT. We revealed that a higher density of the mesopancreas (cut off −9.0 HU) significantly correlated with an actual positive infiltration status of the mesopancreas with a 2.7 -fold increase in chance in the entire cohort of hPDAC patients.

We and others have already studied the feasibility of mesopancreatic fat stranding (MPS) which refers to a presumed increase in density in the mesopancreatic tissue [21–23, 33]. Navez et al. [22] reported that patients exhibiting higher mesopancreatic density, were significantly more likely to have positive resection margins, particularly along the vascular groove. This subgroup also showed significantly worse disease-free and overall survival, further underscoring our thesis that radiographically assessed mesopancreatic characteristics correlate with clinically staging and relevant outcomes. Similarly, Wellner et al. [23] showed that mesopancreatic stromal reaction, defined as tumor-associated stroma at the mesopancreatic margin, can be anticipated on preoperative CT imaging. This concept allowed a further stratification of conventionally defined R0 resections, as patients with residual stromal involvement (S+) had significantly worse overall survival compared with those with complete stromal clearance (S0) (*p = 0.005*).

These findings further demonstrate the clinical relevance of radiographically detectable mesopancreatic tissue alterations. Limitations of all these assessments [20–23] include the subjective evaluation of whether mesopancreatic fat stranding is present on MDCT imaging. In addition, several studies lacked a detailed histopathological assessment of the resected mesopancreatic tissue, thereby limiting a direct correlation between radiographic findings and mesopancreatic infiltration.

To overcome this bias, we applied an objective radiographic approach with the implementation of a reproducible radiographic technique, by determining [1] the density by Hounsfield units and [2] the dimension of the mesopancreas. In our opinion such an analysis is reproducible and may facilitate standardization.

Although mesopancreatic density did not significantly correlate with resection margin status or overall survival in the entire cohort, we were able to demonstrate a clinical relevance in primary resectable hPDAC patients. In this subgroup, increased mesopancreatic density was associated with true MP infiltration and with an increased risk of disease recurrence, suggesting a biologically more aggressive tumor behaviour within the primary resectable cohort. Such findings could suggest further investigations into whether neoadjuvant treatment should be implemented in MP+ primary resectable PDAC patients as well.

We acknowledge that these findings are limited by incomplete follow-up and a relatively short median observation period, which restricts definitive conclusions regarding long-term oncological outcomes. Nevertheless, the observed separation in disease-free survival indicates a consistent trend towards a higher recurrence risk in primary resectable patients with increased mesopancreatic density, compared with those without radiographic mesopancreatic accentuation. Importantly, these observations are concordant with previous reports by Navez et al. [22]. and Wellner et al. [23], who independently demonstrated that radiographically altered mesopancreatic tissue characteristics are associated with impaired oncological outcomes and an increased risk of recurrence. Taken together, these converging findings support the concept that mesopancreatic imaging features capture clinically relevant tumor biology and may allow for a more refined risk stratification within the conventionally defined resectability criteria, warranting further validation in larger cohorts with comprehensive follow-up. Our results provided first insights, since primary resectable hPDAC patients had a similar MP infiltration rate when compared to borderline resectable hPDAC patients.

To complete the analysis in this study, we hypothesized that a smaller anatomical space of the mesopancreas might result in a positive resection status or mesopancreatic infiltration status. We were able to demonstrate that a smaller cranio-caudal extension was associated with a higher risk for R1/R0CRM+ status at the dorsal resection margin and that a decreased horizontal extension towards the SMA in a higher risk for R1/R0CRM+ status at the medial resection margin. A previous study by Endo et al. showed that a thick mesopancreas resulted in a longer operative time, fewer resected lymph nodes and a longer hospital stay [34]. This supports our initially suspected counterintuitive results regarding the dorsoventral extension (thickness of the mesopancreas). A thicker mesopancreas negatively influenced the oncological and histopathological results regarding the resection status (*p = 0.013 and p = 0.006*).

The study has some limitations which have to be considered. First, the retrospective and unicentric nature of the study with an unknown bias; homogenously operated collective cannot be analyzed in a different fashion. Secondly, we did not incorporate the ECOG performance status and CA19-9 values. Nonetheless, the anatomical classification for resectability remains the most significant variable which we have incorporated. Third, no formal interobserver reliability analysis was performed; however, image evaluation was carried out using a predefined, consensus-based approach, which reduced interobserver variability. In order to recommend potential integration of dimension analysis of the mesopancreas into standard radiographic reporting requires further trials with the body habitus, including: BMI adjusted results, sarcopenia and frailty score. Increased CT morphologic density analysis can be caused by other reasons than direct tumor infiltration. Secondary superinfection, fibrosis, chronic pancreatitis as well as paraneoplastic and desmoplastic reactions, are just a few examples. Another limitation is the long-time frame for patient integration into the study with over 10 years. As stated in the supplemental Table 1 the scan quality has only marginally improved over those years. Admittedly, the use of different CT systems over the study period represents a potential limitation of this retrospective analysis. Although imaging protocols were harmonized across scanners, minor variations in acquisition parameters cannot be completely excluded.

It is impossible to find one single parameter that can predict in a preoperative setting the postoperative outcome. To date, we only incorporate one anatomical facet of presumed tumor extension during preoperative risk stratification [7]. However, the three-dimensional structure of the pancreatoduodenectomy resectate is sophisticated and complex. Since we implemented neoadjuvant treatment plans for high-risk hPDAC patients into clinical practice, ``preoperative`` staging became ``pretherapeutic`` staging. We believe that an additional comprehensive analysis of pancreatic cancer and the peripancreatic space, i.e. the mesopancreas, can help the current standards to stratify primary resectable hPDAC patients even more precisely. This may add complementary information to existing staging concepts and warrants further investigation with regard to its potential implications for neoadjuvant treatment strategies.

## Conclusion

The mesopancreas is an important peripancreatic region which secures the dorsal resection margin. Current resectability criteria do not acknowledge the mesopancreas and thus all facets of tumor extension. Our predefined radiographic parameters predicted histopathological confirmed mesopancreatic infiltration and correlated with less favorable resection status and therefore may warrant inclusion in standardized radiographic reporting.

## Supplementary Information

Below is the link to the electronic supplementary material.

Supplementary Material 1

Supplementary Material 2

Supplementary Material 3

Supplementary Material 4

Supplementary Material 5

Supplementary Material 6

Supplementary Material 7

Supplementary Material 8

Supplementary Material 9

Supplementary Material 10

Supplementary Material 11

## Data Availability

The datasets used and/or analyzed during the current study are available from the corresponding author on reasonable request.

## Abbreviations

AA: Abdominal artery
CRM: Circumferential resection margin
HCC: Hepatocellular carcinoma
HU: Hounsfield units
IVC: Inferior vena cava
MDCT: Multi–detector computed tomography
MP: Mesopancreas
MPE: Mesopancreatic excision
NCCN: National comprehensive cancer network
hPDAC: Pancreatic ductal adenocarcinoma of the pancreatic head
PV: Portal vein
SMA: Superior mesenteric artery
SMV: Superior mesenteric vein
TC: Truncus coeliacus
TNM: Tumor–nodes–metastasis classification
