# Correction: Standardizing computed tomographic assessment of the mesopancreas in pancreatic cancer patients

**DOI:** 10.1007/s00261-026-05656-1

**Published:** 2026-07-02

**Authors:** Stephan David, Sami Safi, Bijan Fink, Iskender Pustu, Sascha Vaghiri, Ahmad Sultani, Andrea Alexander, Michael Wolf-Vollenbroeker, Lena Haeberle-Graser, Christoph Roderburg, Irene Esposito, Wolfram Knoefel, Farid Ziayee

**Affiliations:** Heinrich Heine University Düsseldorf, Düsseldorf, Germany

**Correction to: Abdominal Radiology**

**https://doi.org/10.1007/s00261-026-05486-1**

In this article the Figs. 1, 2, 3, 4, and 5 was missing in the pdf version; the figures should have appeared as shown below.

The original article has been corrected.

**Fig. 1:**
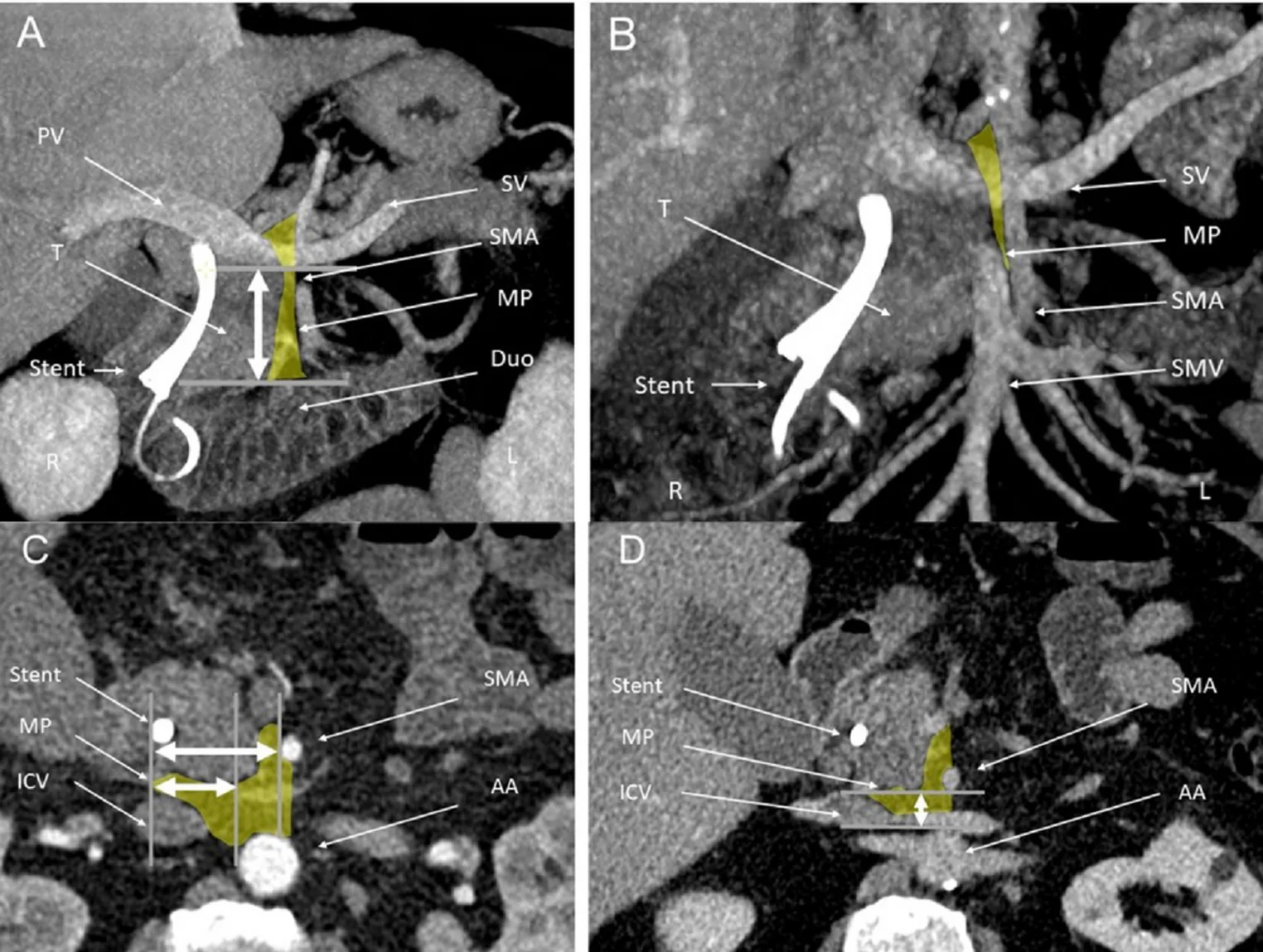
CT-scan measurements provide a standardized approach for quantitatively evaluating mesopancreatic dimensions and density using preoperative imaging: **A** Cranio-caudal extension in coronary layer: Measured from the caudal border of the portal vein to the cranial border of the pars horizontalis duodeni, tangential to the medial border of the pars descendens duodeni (double arrowed line). **B** Coronary visualization of the mesopancreas in reference to the major peripancreatic vessels. Note the anatomical space between the pancreatic tissue and the SMA (yellow hatched area) **C** Horizontal-SMA-extension in transversal CT layer. (1) Horizontal-SMA extension: Measured from the medial border of the pars descendens duodeni to the right border of the superior mesenteric artery, in the last axial layer of the pars descendens duodeni (double arrowed line). Horizontal-AA extension: From the medial border of the pars descendens duodeni to the right border of the abdominal aorta (double arrowed line). **D** Dorso-ventral-tumor extension and Hounsfield measurement of the pancreas in transversal CT layer: Between the dorsal edge of the pancreatic head and the abdominal aorta/inferior caval vein with an auxiliary line, in the layer with the greatest tumor extension (double arrowed line). Furthermore measurements of the Hounsfield unit density in the mesopancreas (yellow hatched area): The average density of the mesopancreas, measured in Hounsfield units, was assessed across the entire region at the greatest pancreatic head extension in the control group or at the level of maximum tumor extension in the hPDAC group. (*Duo* duodenum, *MP* mesopancreas (yellow hatched area), *PV* portal vein, *SMA* superior mesenteric artery, *SMV* superior mesenteric vein, *SV* splenic vein, *T* tumor; grey lines = auxiliary lines; double arrowed line = measured extension)

**Fig. 2:**
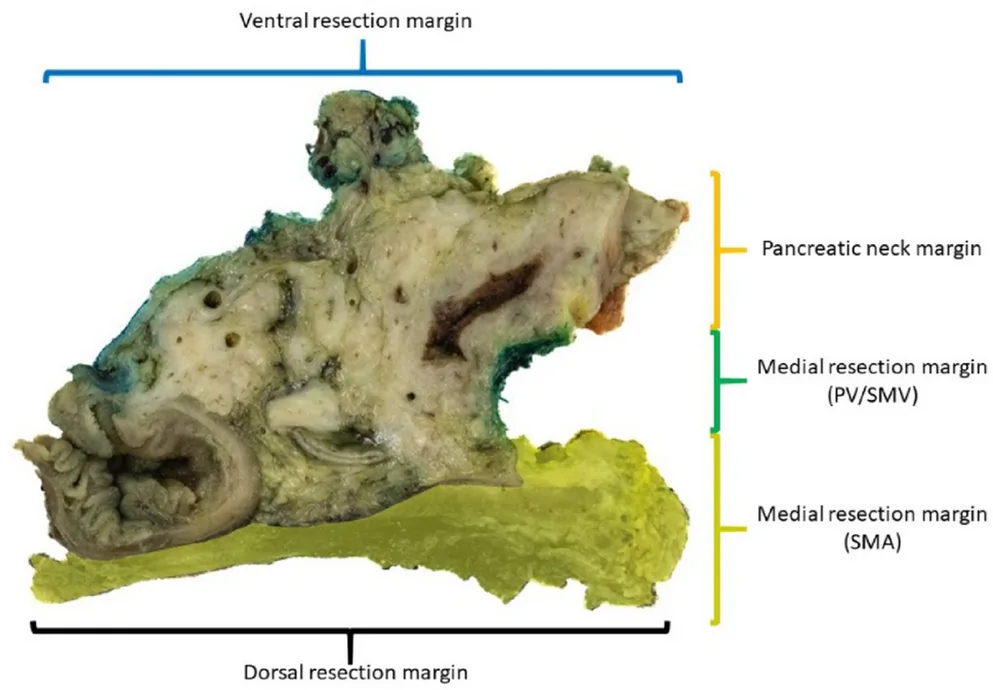
The pathological specimen after pancreatoduodenectomy exhibits invasion into the adjacent peripancreatic adipose tissue (pT2N1L1V1Pn1R0CRM−). To aid in accurate anatomical delineation, the specimen was meticulously marked using a standardized color-coding protocol (dorsal margin = black; ventral margin = blue; medial PMV/SMV margin = green; medial SMA margin = yellow; mesopancreas = yellow hatched area). Macroscopic examination was performed following the axial slicing method ensuring a thorough and methodical assessment of the tissue structure

**Fig. 3:**
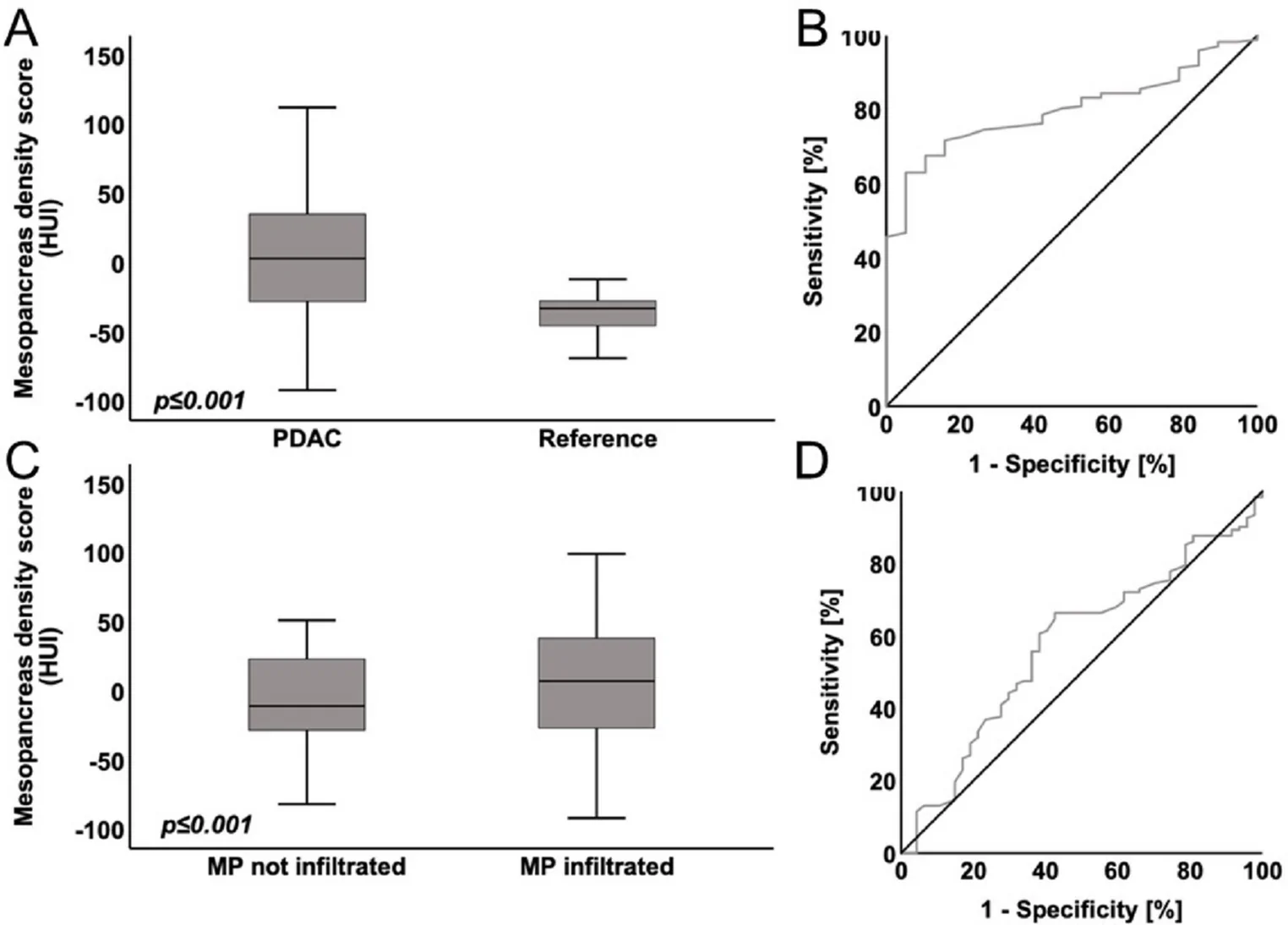
**A** Box plot analysis of radiographically assessed mesopancreatic density in hPDAC patients vs. reference group. Mann-Whitney U test was used to test for statistical difference between these groups. **B** ROC analysis for mesopancreatic density, hPDAC patients vs. reference group. **C** Box plot analysis of radiographically assumed mesopancreatic density in relation to the true mesopancreatic infiltration status. Mann-Whitney U test was used to test for statistical significance. **D** ROC analysis for mesopancreatic density vs. true mesopancreatic infiltration status in hPDAC patients

**Fig. 4:**
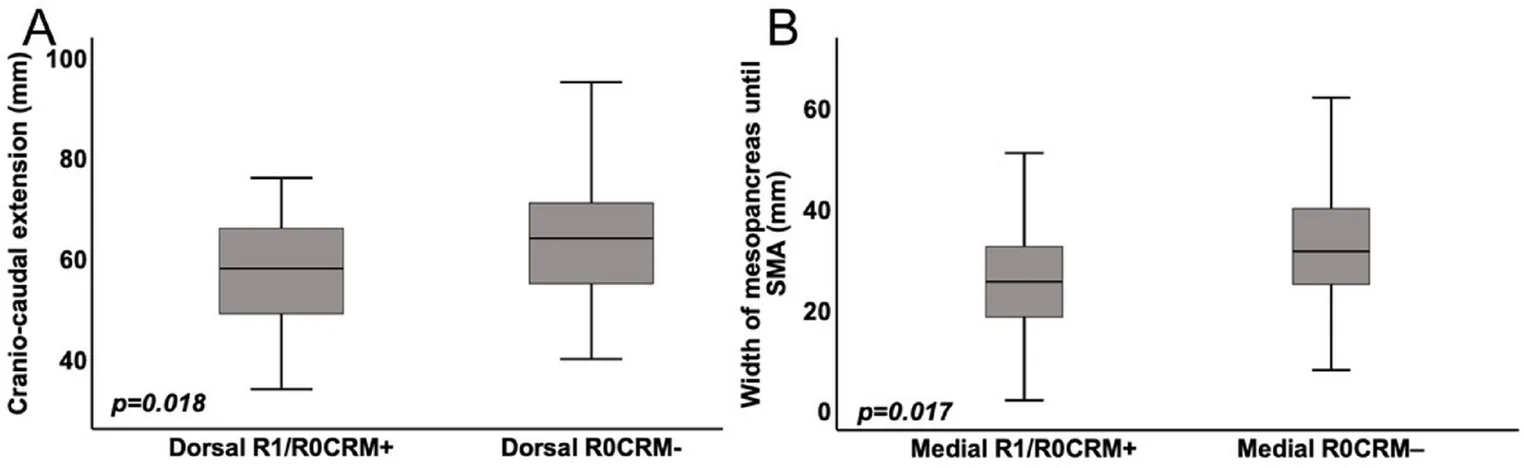
**A** Box plot analysis of radiographically assessed cranio-caudal extension of the mesopancreas vs. dorsal resection margin status. **B** Box plot analysis of radiographically assessed horizontal-SMA extension of the mesopancreas vs. medial resection margin status. Mann-Whitney U test was used to test for statistical significance

**Fig. 5:**
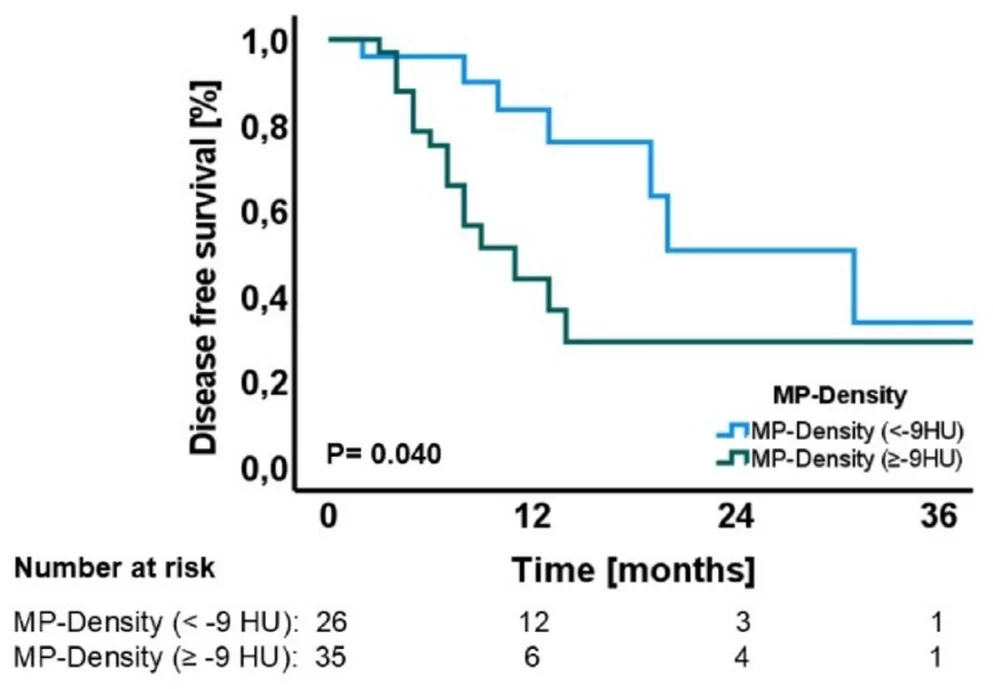
Kaplan–Meier analysis of disease-free survival (DFS) in primary resectable hPDAC patients stratified by mesopancreatic (MP) density on preoperative MDCT using the predefined cut-off of −9 HU. Patients with reduced MP density (< − 9 HU) showed significantly longer DFS compared with those with increased MP density (≥ − 9 HU) (*p = 0.040*). Numbers at risk are shown below the plot

