# Supplementary material for: Standardizing computed tomographic assessment of the mesopancreas in pancreatic cancer patients
Source: Abdom Radiol (NY). 2026 Apr 9;51(10):5058–69. doi: 10.1007/s00261-026-05486-1 (PMC13577993; doi:10.1007/s00261-026-05486-1)
Supplement: Supplementary file 2 [file 261_2026_5486_MOESM11_ESM.docx]

| **Supplemental Table 3:**  Prediction analysis of the Mesopancreatic infiltration status by radiographically measured mesopancreatic density (HU) in hPDAC patients and the reference group. Optimal cut-off point for density was computed by ROC-analysis. Ordinal scaling of density (<Cut-off and > Cut-off) was tested for distribution against MP infiltration status (Chi-Square test). Binary logistic regression analysis was used for prediction analysis | | | | | | | |
| --- | --- | --- | --- | --- | --- | --- | --- |
| **Optimal Cut off by ROC Analysis** | | | −11.5 HUI | | | | |
|  | | | | | | | |
|  | MP Infiltration status | | | | | ***p-value*** | |
|  | Reference Group | hPDAC | | | | ***<0.001*** | |
| **MP density**  **<−11.5HU** | 18 | 64 | | | | ***Sensitivity*** | |
|  |  |  |  |  |  | *94.7%* | |
|  |  |  |  |  |  | ***Specificity*** | |
|  |  |  |  |  |  | *63.0%* | |
| **MP density**  **≥**−**11.5HU** | 1 | 109 | | | | ***PPV*** | |
|  |  |  |  |  |  | *21.9%* | |
|  |  |  |  |  |  | ***NPV*** | |
|  |  |  |  |  |  | *99.1%* | |
|  | | | | | | | |
|  | | | | p-value | Odds Ratio | | 95% CI |
| **Binary logistic Regression analysis** | | | | ***<0.001*** | 30.7 | | 4.0 – 235.1 |
