# Supplementary material for: Standardizing computed tomographic assessment of the mesopancreas in pancreatic cancer patients
Source: Abdom Radiol (NY). 2026 Apr 9;51(10):5058–69. doi: 10.1007/s00261-026-05486-1 (PMC13577993; doi:10.1007/s00261-026-05486-1)
Supplement: Supplementary file 3 [file 261_2026_5486_MOESM1_ESM.docx]

| **Supplemental Table 5:**  Prediction analysis of the Mesopancreatic infiltration status by current resectability criteria in hPDAC patients. Ordinal scaling of resectability (primary resectable vs. borderline resectable) was tested for distribution against MP infiltration status (Chi-Square test). Binary logistic regression analysis was used for prediction analysis. | | | | | | |
| --- | --- | --- | --- | --- | --- | --- |
|  | MP Infiltration status | | | | ***p-value*** | |
|  | MP not infiltrated | MP infiltrated | | | *0.342* | |
| **Primary resectable** | 37 | 87 | | | ***Sensitivity*** | |
|  |  |  |  |  | *29.66%* | |
|  |  |  |  |  | ***Specificity*** | |
|  |  |  |  |  | *77.78%* | |
| **Borderline resectable** | 11 | 38 | | | ***PPV*** | |
|  |  |  |  |  | *77.78%* | |
|  |  |  |  |  | ***NPV*** | |
|  |  |  |  |  | *29.66%* | |
|  | | | | | | |
|  | | | p-value | Odds Ratio | | 95% CI |
| **Binary logistic Regression analysis** | | | *0.344* | 1.5 | | 0.6 – 3.3 |
