# Supplementary material for: Standardizing computed tomographic assessment of the mesopancreas in pancreatic cancer patients
Source: Abdom Radiol (NY). 2026 Apr 9;51(10):5058–69. doi: 10.1007/s00261-026-05486-1 (PMC13577993; doi:10.1007/s00261-026-05486-1)
Supplement: Supplementary file 4 [file 261_2026_5486_MOESM2_ESM.docx]

| **Supplemental Table 4:**  Correlation analysis between preoperative resectability status, and geometric-extension data as well as MP density within in the hPDAC group. Current resectability criteria are implemented from the national comprehensive cancer network (NCCN). Statistical significance was calculated using the Mann-Whitney U test. (*p≤0.05* is significant). | | | |
| --- | --- | --- | --- |
|  | **Primary resectable**  n= 124 | **Borderline resectable**  n=49 |  |
|  | median (min.-max.) | median (min.-max.) | ***p-value*** |
| **Cranio- caudal extent (mm)** | 58.0 (34.0-95.0) | 66.0 (45.0-95.0) | *0.100* |
| **Horizontal-SMA-extent (mm)** | 30.0 (8.0-63.0) | 27.0 (2.0-48.0) | *0.325* |
| **Horizontal-AA-extent**  **(mm)** | 35.0 (9.0-67.0) | 40.0 (18.0-71.0) | *0.057* |
| **Dorso-ventral-extent-PV**  **(mm)** | 13.0 (2.0-28.0) | 11.0 (2.0-21.0) | *0.117* |
| **Dorsal-ventral-T-extent- (mm)** | 8.0 (2.0-28.0) | 5.0 (2.0-23.0) | *0.163* |
| **Density (HU)** | -6 (-92-112) | 13.0 (-70-99) | *0.163* |
