# Supplementary material for: Standardizing computed tomographic assessment of the mesopancreas in pancreatic cancer patients
Source: Abdom Radiol (NY). 2026 Apr 9;51(10):5058–69. doi: 10.1007/s00261-026-05486-1 (PMC13577993; doi:10.1007/s00261-026-05486-1)
Supplement: Supplementary file 5 [file 261_2026_5486_MOESM3_ESM.docx]

| **Supplemental Table 7:**  Correlation analysis between Mesopancreas infiltration status and geometric-extension data as well as MP density within in the 49 borderline resectable hPDAC group. Statistical significance calculated using the Mann-Whitney U test. (*p≤0.05* is significant). | | | |
| --- | --- | --- | --- |
|  | **Mesopancreas**  **infiltrated**  n= 38 | **Mesopancreas**  **not infiltrated**  n=11 |  |
|  | median (min.-max.) | median (min.-max.) | ***p-value*** |
| **Cranio- caudal extent (mm)** | 59.0 (40.0-90.0) | 65.0 (34.0-95.0) | *0.420* |
| **Horizontal-SMA-extent (mm)** | 29.0 (7.0-63.0) | 31.0 (2.0-54.0) | *0.689* |
| **Horizontal-AA-extent**  **(mm)** | 36.0 (16.0-67.0) | 37.0 (9.0-71.0) | *0.420* |
| **Dorso-ventral-extent-PV**  **(mm)** | 13.0 (2.0-28.0) | 11.0 (4.0-28.0) | *1.000* |
| **Dorsal-ventral-T-extent- (mm)** | 8.0 (2.0-26.0) | 6.0 (2.0-230.) | *1.000* |
| **Density (HU)** | 7.0 (−92.0-99.0) | −11.0 (−82.0-112.0) | *0.780* |
