# Supplementary material for: Standardizing computed tomographic assessment of the mesopancreas in pancreatic cancer patients
Source: Abdom Radiol (NY). 2026 Apr 9;51(10):5058–69. doi: 10.1007/s00261-026-05486-1 (PMC13577993; doi:10.1007/s00261-026-05486-1)
Supplement: Supplementary file 6 [file 261_2026_5486_MOESM4_ESM.docx]

| **Supplemental Table 9:**  Correlation analysis between dorsal R-Status and geometric-extension data as well as MP density within in the borderline resectable hPDAC-Group. Statistical significance was calculated using the Mann-Whitney U test. (*p≤0.05* is significant). | | | |
| --- | --- | --- | --- |
|  | **Dorsal**  **R0CRM**− | **Dorsal**  **R1/R0CRM+** |  |
|  | median (min.-max.) | median (min.-max.) | ***p-value*** |
| **Cranio- caudal extent (mm)** | 64.0 (40.0-95.0) | 58.0 (34.0-76.0) | *0.409* |
| **Horizontal-SMA-extent (mm)** | 30.0 (2.0-63.0) | 30.0 (10.0-56.0) | *0.181* |
| **Horizontal-AA-extent**  **(mm)** | 35.5 (9.0-71.0) | 40.5 (9.0-60.0) | *0.672* |
| **Dorso-ventral-extent-PV**  **(mm)** | 11.0 (2.0-28.0) | 14.5 (2.0-28.0) | ***0.018*** |
| **Dorsal-ventral-T-extent- (mm)** | 6.0 (2.0-26.0) | 9.0 (2.0-26.0) | ***0.011*** |
| **Density (HU)** | 4.0 (−85.0-112.0) | −6.0 (−92.0-98.0) | *0.685* |
