# Supplementary material for: Standardizing computed tomographic assessment of the mesopancreas in pancreatic cancer patients
Source: Abdom Radiol (NY). 2026 Apr 9;51(10):5058–69. doi: 10.1007/s00261-026-05486-1 (PMC13577993; doi:10.1007/s00261-026-05486-1)
Supplement: Supplementary file 7 [file 261_2026_5486_MOESM5_ESM.docx]

**Supplemental Table 1**: Scanner specifications and radiation dose metrics for the three CT systems included in this study. Values for CTDI𝑣𝑜𝑙 and DLP are reported as median for each acquisition step, including topogram, pre-monitoring, monitoring, arterial phase, and portal-venous phase. All scanners used standard iterative reconstruction and automated tube current modulation (CARE Dose 4D)

| **Parameter** | **Definition Flash** | **SOMATOM Edge** | **Definition AS (Sliding Gantry)** |
| --- | --- | --- | --- |
| **Rows** | 2×128 | 128 | 64 |
| **Reconstruction slice thickness** | 2 mm | 2 mm | 2 mm |
| **CARE Dose 4D** | Yes | Yes | Yes |
| **Topogram CTDIvol (mGy)** | 0.14 [0.12–0.16] | 0.15 [0.13–0.17] | 0.15 [0.13–0.17] |
| **Topogram DLP (mGy·cm)** | 10 [9–12] | 10 [9–12] | 10.7 [9.5–12.0] |
| **Pre-monitor CTDIvol (mGy)** | 1.14 [1.0–1.3] | 1.2 [1.0–1.3] | 1.25 [1.1–1.4] |
| **Pre-monitor DLP (mGy·cm)** | 1.0 [0.9–1.2] | 1.1 [0.9–1.2] | 1.3 [1.1–1.5] |
| **Monitoring CTDIvol (mGy)** | 10.3 [9.0–11.5] | 9.5 [8.5–10.5] | 8.8 [7.9–9.7] |
| **Monitoring DLP (mGy·cm)** | 10 [9–12] | 9.5 [8.5–10.5] | 8.8 [7.9–9.7] |
| **Upper-abdomen arterial CTDIvol (mGy)** | 3.9 [3.4–4.3] | 3.7 [3.3–4.1] | 3.8 [3.3–4.2] |
| **Upper-abdomen arterial DLP (mGy·cm)** | 100 [90–110] | 90 [80–100] | 59 [53–66] |
| **Portal-venous CTDIvol (mGy)** | 6.7 [6.0–7.4] | 6.3 [5.8–7.0] | 5.5 [5.0–6.1] |
| **Portal-venous DLP (mGy·cm)** | 450 [410–500] | 430 [390–480] | 366 [330–405] |
| **Total DLP (mGy·cm)** | 520 [470–580] | 500 [450–560] | 460 [420–510] |
