# Supplementary material for: Standardizing computed tomographic assessment of the mesopancreas in pancreatic cancer patients
Source: Abdom Radiol (NY). 2026 Apr 9;51(10):5058–69. doi: 10.1007/s00261-026-05486-1 (PMC13577993; doi:10.1007/s00261-026-05486-1)
Supplement: Supplementary file 10 [file 261_2026_5486_MOESM8_ESM.docx]

| **Supplemental Table 2:**  Correlation analysis of geometric-extension data and density between the hPDAC-Group and Reference-Group. Statistical significance calculated using the Mann-Whitney U Test. (*p≤0.05* is significant). | | | |  |
| --- | --- | --- | --- | --- |
|  | **hPDAC**  **n=173** | **Reference**  **n=19** |  | |
|  | median (min.-max.) | median (min.-max.) | ***p-value*** | |
| **Cranio- caudal extent (mm)** | 61.5 (34.0-95.0) | 62.0 (39.0-85.0) | *0.545* | |
| **Horizontal-SMA-extent (mm)** | 30.0 (2.0-63.0) | 36.0 (15.0-55.0) | *0.059* | |
| **Horizontal-AA-extent**  **(mm)** | 37.0 (9.0-71.0) | 39.0 (13.0-55.0) | *0.368* | |
| **Dorso-ventral-PV- extent-**  **(mm)** | 12.0 (2.0-28.0) | 13.0 (7.0-23.0) | *0.502* | |
| **Dorsal-ventral-T-extent- (mm)** | 7.0 (2.0-26.0) | 9.0 (3.0-14.0) | *0.472* | |
| **Density- (HU)** | 3.0 (-92.0-112.0) | -33.0 (-82.0-7.0) | ***<0.001*** | |
