# Supplementary material for: Standardizing computed tomographic assessment of the mesopancreas in pancreatic cancer patients
Source: Abdom Radiol (NY). 2026 Apr 9;51(10):5058–69. doi: 10.1007/s00261-026-05486-1 (PMC13577993; doi:10.1007/s00261-026-05486-1)
Supplement: Supplementary file 11 [file 261_2026_5486_MOESM9_ESM.docx]

| **Supplemental Table 10:**  Correlation analysis between R-Status of the vascular groove (medial CRM), and geometric-extension data as well as MP density within in primary resectable hPDACs. Statistical significance was calculated using the Mann-Whitney U test. (*p≤0.05* is significant). | | | |
| --- | --- | --- | --- |
|  | **Vascular groove**  **R0CRM**− | **Vascular groove**  **R1/R0CRM+** |  |
|  | median (min.-max.) | median (min.-max.) | ***p-value*** |
| **Cranio- caudal extent (mm)** | 60.5 (34-95) | 65 (45-95) | *0.573* |
| **Horizontal-SMA-extent (mm)** | 32 (8-62) | 26.5 (2-63) | *0.112* |
| **Horizontal-AA-extent**  **(mm)** | 37 (9-65) | 38.5 (12-71) | *0.275* |
| **Dorso-ventral-extent-PV**  **(mm)** | 13 (2-28) | 12 (9-71) | *0.120* |
| **Dorsal-ventral-T-extent- (mm)** | 6 (2-26) | 6.5 (2-26) | *0.371* |
| **Density (HU)** | −2 (−85-112) | 8.0 (−92-51) | *0.782* |
